# A study on the therapeutic effect of precise clipping of intracranial aneurysms assisted by CTA and 3D-slicer software

**DOI:** 10.3389/fsurg.2025.1535585

**Published:** 2025-02-17

**Authors:** Chuan He, Gang Cao, Ying Yang, Qi Zhong, Yongsheng Lei, Xingshi Tan, Xiaohong Lin, Yaokun Chen, Wenxiao Sun

**Affiliations:** Department of Neurosurgery, Zhuhai City Hospital of Integrated Traditional Chinese and Western Medicine (Zhuhai Hospital Affiliated with Southern Medical University, China), Zhuhai, China

**Keywords:** intracranial aneurysm, 3D-slicer software, CTA, microsurgical clipping, micro-neurosurgery

## Abstract

**Objective:**

To evaluate the application of Computed Tomography Angiography (CTA) combined with 3D-Slicer software reconstruction technology in the diagnosis and preoperative planning of intracranial aneurysms, and to explore its positive significance in improving surgical outcomes for patients.

**Methods:**

From January 2021 to December 2023, a total of 24 patients diagnosed with intracranial aneurysms (25 cases) underwent craniotomy clipping surgery. We utilized 3D-Slicer to render and fuse preoperative CTA image data, and to formulate individualized surgical plans, including approach, bone window, anticipated protection of the aneurysm-bearing artery and perforating arteries, and compared these with the actual surgical procedures. The actual intraoperative conditions were used as the diagnostic reference standard to compare and analyze the morphology of intracranial aneurysms, assessing the consistency between preoperative planning and actual operations. Additionally, intraoperative and postoperative complications and prognosis were analyzed.

**Results:**

It was confirmed that among the 25 intracranial aneurysms, the combination of CTA and 3D-Slicer could effectively detect and reconstruct these aneurysms; quantifying the extent of bone window grinding could reduce the need for multiple bone grinding after opening the dura mater; the consistency in determining the stenosis of the aneurysm-bearing artery intraoperatively, the difficulty of reconstructing and separating the aneurysm neck, and the condition of the perforating arteries near the aneurysm was good, with Kappa values of 0.865, 0.779, and 0.635, respectively. However, the consistency in predicting the rupture orientation of the aneurysm was poor, with a Kappa value of 0.186. All aneurysms in this group were completely clipped, and no new signs of bleeding were found in the head CT within 24 h after surgery, and no signs of aneurysm recurrence were observed in the head CTA within 7 days.

**Conclusion:**

In summary, combining 3D-Slicer technology with CTA can accurately assess intracranial aneurysms and provide key anatomical information required for craniotomy clipping surgery to formulate surgical plans, which has a positive significance in reducing surgical complications. These findings not only lay the foundation for further exploration of related issues but also provide clinical doctors with more scientifically effective guidance for diagnosis and surgical planning.

## Introduction

Intracranial aneurysms (IAs) can lead to rapid progression and life-threatening conditions upon rupture and hemorrhage. The primary treatment options are microsurgical clipping and endovascular embolization. The choice of treatment largely depends on the patient's age, the speed of disease progression, the location and size of the aneurysm, the condition of the aneurysm-bearing artery and perforating branches, and the presence of complications such as intracranial hematoma or even brain herniation (1). Microsurgical clipping offers advantages such as a lower long-term recurrence rate and the ability to clear hematomas simultaneously, maintaining a significant advantage for middle cerebral artery aneurysms despite the development of interventional treatments. Preoperative assessment of intracranial vascular conditions currently involves Computed Tomography Angiography (CTA), Magnetic Resonance Angiography (MRA), and Digital Subtraction Angiography (DSA), each with its own strengths and weaknesses. Clinical physicians tend to prioritize practicality in examinations, focusing not only on diagnosis and providing simple axial, coronal, and sagittal image data but also on the utility of the examination. This differs from the perspective of radiologists, who typically handle reconstructions using imaging workstations in radiology departments, diagnosing hematomas and vascular reconstructions, which occasionally leads to missed or misdiagnoses. For surgeons, it is crucial to understand the three-dimensional images of the aneurysm from different angles and surgical positions, combined with vascular reconstruction of the skull base, brain tissue, and hematoma, to fully grasp the morphological characteristics of the aneurysm, the form and position of the aneurysm-bearing artery, and its relationship with surrounding tissue structures (2). Therefore, seeking a new method for reconstructing imaging data of IAs is of great significance, primarily for rapid diagnosis of IAs (especially those close to the skull base, with hematoma, or recurring after intervention or clipping surgery), and secondly, for planning subsequent surgical procedures to assist in the precise clipping surgery of IAs. To this end, this study utilizes CTA, the most widely used clinical examination and the most easily conducted pre-emergency surgery, in conjunction with the post-image processing software 3D-Slicer. It fuses and reconstructs imaging data of patients with IAs suitable for microsurgical clipping treatment, analyzes aneurysm characteristics, and plans precise surgical strategies for subsequent aneurysm clipping, attempting to explore the significance of this technology in diagnosis and treatment.

## Materials and methods

### Ethical statement

This study was approved by the Medical Ethics Committee of Zhuhai Hospital of Integrated Traditional Chinese and Western Medicine (Ethics No: EC-C-008-A04-V1.0), all research was performed in accordance with relevant guidelines and regulations, and each family member of the surgical patients signed an informed consent form and was fully informed about the treatment process.

### Patient cohort

A total of 24 patients with intracranial aneurysms admitted to the Department of Neurosurgery at Zhuhai Hospital of Integrated Traditional Chinese and Western Medicine from January 2021 to December 2023 were selected. All patients experienced subarachnoid hemorrhage due to the rupture of an aneurysm. Among them, there were 7 males and 17 females, aged 41–73 years old, with an average age of (54.52 ± 8.87) years. A total of 25 aneurysms were identified in this group of patients. Based on the CTA imaging data after admission, precise surgical plans were made following the fusion rendering and three-dimensional reconstruction using the 3D-Slicer software, followed by microsurgical clipping surgery under a microscope. Inclusion criteria: the timing of surgery was within 1–48 h after onset; patients who had undergone CTA examination after admission and had a clear diagnosis of intracranial aneurysm; patients who met the indications for clipping surgery for intracranial aneurysms, with aneurysms located in the posterior communicating segment of the internal carotid artery, the middle cerebral artery and its branches, and the anterior communicating artery; patients with multiple aneurysms or recurrent aneurysms, but with a clear responsible aneurysm suitable for clipping surgery; patients whose family members provided informed consent and signed an informed consent form. Exclusion criteria: patients with clear contraindications for craniotomy clipping surgery, including systemic diseases or severe systemic diseases that cannot undergo craniotomy surgery; patients with clear intracranial aneurysms but with other cerebrovascular diseases that affect the evaluation of research efficacy; patients or family members who refuse craniotomy surgery or cannot undergo follow-up treatment; patients with posterior circulation aneurysms who should preferentially undergo interventional treatment. All patients and family members have signed consent forms, they consented to the publication of their photos (Table 1).

**Table 1:** General information and intracranial aneurysm data of the patients in this series.

Project indicators		Numerical data
Age		54.52 ± 8.87 years
Sex	Male	7 (41.2%)
	Female	17 (58.8%)
Total		24 individuals
Aneurysm location	Anterior communicating artery	6 (24%)
	Posterior communicating artery	4 (16%)
	Middle cerebral artery	15 (60%)
Total		25 cases
Maximum aneurysm diameter		8.28 ± 4.40 mm
Aneurysm height		6.49 ± 3.77 mm
Aneurysm width		5.53 ± 3.42 mm
Aneurysm neck width		4.56 ± 2.42 mm

### Equipment

Siemens SOMATOM Drive CT, Zeiss K900 microscope, personal computer running 3D-Slicer software.

### Fusion rendering and three-dimensional reconstruction

All patients underwent preoperative head and neck CTA examinations in the radiology department using a Siemens SOMATOM Drive CT. (dual-source 128-slice, slice thickness 0.75 mm). The patients' original DICOM data were imported into 3D-Slicer version 4.13.0-2022-02-19.

Firstly, the Volume Rendering module was used to fuse and render the details of the skull and intracranial vessels, adjusting the “Volume properties” option in “Scalar Opacity Mapping” to restore the patients' anatomical relationships, identify artifacts from the original embolization and clipping materials of intracranial hematomas and recurrent aneurysms, and fully display the flow arteries to understand the presence of any stenosis, details of the aneurysm sac, and possible rupture sites, as well as the relationship with surrounding perforating arteries.

Aneurysm data were measured, and then surgical approaches were selected based on the diagnosis of the aneurysm, and surgical plans were made, including the angles of deflection and retroflexion, the range of the bone window, the extent of skull base bone removal, the order of intraoperative angiogram exposure, and the selection of clipping methods and directions.

The Image Segmentation module (Segment Editor) was selected, using thresholds and stereo drawing to generate images of the skull, intracranial vessels, intracranial aneurysms, hematomas, brain tissue, etc., as needed, with a particular focus on the impact on the flow arteries and perforating arteries during the clipping process from the surgical angle.

Two physicians from a fixed imaging diagnosis panel and two surgeons from a fixed surgical treatment team were selected. The imaging assessment was jointly conducted by the same attending neurosurgeon with over 15 years of experience and the same attending radiologist with over 15 years of experience. Preoperative and intraoperative evaluations were made separately for points of interest, with a comparison between preoperative planning and the actual intraoperative situation.

### Clipping surgery procedure

The surgeries of the selected group of patients were all performed by the same chief physician with over 15 years of experience in the neurosurgery field, and the assistant was also a deputy chief physician with more than 15 years of experience in the same specialty. Based on the preoperative plan, a pterional approach or an extended pterional approach was used, and in one case of the anterior communicating artery, a subfrontal approach through the interhemispheric fissure was adopted according to the situation. The Mayfield head frame was used for fixation to maintain the planned head position and deflection angle, and the planned range of the bone window and the removal range from the sphenoid ridge to the subfrontal bone were opened as planned. During the surgery, the lateral fissure and carotid cistern were fully opened, lamina terminalis fenestration, and cerebrospinal fluid was slowly released for adequate exposure. Depending on the location of the aneurysm, the middle cerebral artery, internal carotid artery, and posterior communicating artery, as well as the anterior cerebral artery and anterior communicating artery were exposed, and the aneurysm-bearing artery was displayed preoperatively according to the plan, the neck of the aneurysm was separated, and the perforating arteries were exposed with care taken to protect them. Indocyanine green fluorescence angiography was performed before clipping, and the aneurysm-bearing artery was temporarily occluded to reduce the tension of the aneurysm sac, and the neck of the aneurysm was separated again to confirm the clipping position and angle, and photographs were taken for comparison with the preoperative plan. The aneurysm clip was adjusted until the aneurysm no longer appeared on the fluorescence angiography, ensuring that the aneurysm-bearing artery and perforating arteries were well visualized. Papaverine (60 mg) was dissolved in 20 ml of normal saline and gently irrigated in the surgical field, and a wet gelatin sponge containing papaverine was applied to the aneurysm-bearing artery to prevent cerebral vasospasm. The dura mater was tightly sutured, and the bone flap was replaced or removed for decompression as needed based on the condition. Photographs were taken before and after clipping for comparison with the preoperative plan to evaluate the role of precise surgical planning in facilitating the operation and reducing intraoperative complications.

### Evaluation indicators

The characteristics of the aneurysm preoperative planning were analyzed using SPSS 23.0; the difficulty in reconstructing and separating the aneurysm neck, the prediction of the rupture orientation of the aneurysm, the prediction of the condition of the perforating arteries near the aneurysm, and the prediction of the stenosis of the aneurysm-bearing artery were compared with the actual intraoperative conditions and subjected to Kappa consistency testing.

## Results

### Preoperative findings revealed that the comparison between three-dimensional reconstructed images of aneurysms and intraoperative observations yielded the following results

Fifteen cases of middle cerebral artery aneurysms and six cases of anterior communicating artery aneurysms, along with four cases of posterior communicating artery aneurysms, were identified through preoperative CTA data reconstructed using Slicer software. All were true saccular aneurysms, with one case of an anterior communicating artery aneurysm being a recurrent rupture after endovascular intervention. Among these, 18 cases presented with intracranial hematomas associated with aneurysms, and seven cases had pure subarachnoid hemorrhage. In terms of morphological characteristics, ten cases exhibited a protruding daughter sac on the aneurysm's surface. The diagnosis of aneurysms, the orientation of the aneurysm sac, the relationship between the aneurysm and the hematoma, and the course of the parent artery were clearly determined by comparing intraoperative observations with preoperative predictions.

Comparing the preoperative 3D Slicer reconstruction results with those from CTA reconstruction, the rendering clearly distinguished the skull and artifacts better than the CTA reconstructions produced by the imaging workstation. The reconstruction also included hematomas and brain parenchyma, allowing for the simulation of craniotomy and bone window placement, as well as the positioning of body angles, providing more effective information to assist in surgery.

### Surgical outcomes: comparison of aneurysm characteristics with intraoperative findings and clipping status

Based on preoperative reconstructions, intraoperative planning was refined to reduce unnecessary opening of the dura and further removal of the skull base after the initial craniotomy, allowing for a more strategic approach to the passage of the clip and the extent of the hematoma. In this series, all aneurysms were completely clipped, with intraoperative fluorescence angiography confirming no occlusion of the parent artery or surrounding vessels at the neck of the aneurysm.

Exposure of the aneurysm and analysis of its characteristics revealed a comparison between preoperative predictions and intraoperative observations. Two cases confirmed preoperative predictions of difficulty in dissecting the neck due to adhesions and hematoma encapsulation, while 22 cases were easier to separate as anticipated (Table 2). One case, anticipated to be challenging intraoperatively, was found to be a metallic artifact. In terms of aneurysm rupture site determination, 18 cases matched preoperative reconstruction predictions, while in 7 patients, the rupture site was either not predictable or identified intraoperatively as a hematoma-encapsulated daughter sac or multiple daughter sacs, which were not the source of the current hemorrhage (Table 3). Regarding the preoperative assessment of perforating arteries near the aneurysm, there was a concordance between preoperative and intraoperative findings in 21 cases. In three cases, no significant perforating arteries were identified preoperatively, but small arteries closely adhering to the aneurysm wall were discovered intraoperatively. In one case, an arterial shadow adjacent to the aneurysm neck identified preoperatively was confirmed to be an artifact intraoperatively (Table 4). The preoperative judgment of potential stenosis in the parent artery was accurate in 24 cases. In one patient, stenosis due to middle cerebral artery atherosclerosis was anticipated, but intraoperatively, it was found to be related to compression by a hematoma in the sylvian fissure. After evacuating the hematoma and clipping the aneurysm, no stenosis was observed in the parent artery (Table 5). The preoperative prediction of stenosis in the parent artery showed excellent agreement with intraoperative findings (Kappa value >0.85, *P* < 0.001). The preoperative estimation of the difficulty in dissecting the aneurysm neck and the presence of perforating arteries near the aneurysm showed good agreement (Kappa values >0.6, *P* < 0.001), while the preoperative prediction of the rupture site orientation of the aneurysm showed poor agreement (Kappa value 0.186, less than 0.45, *P* = 0.349).

**Table 2:** Preoperative reconstruction images predict the difficulty of aneurysm neck separation and compare with the actual situation during surgery.

		Intraoperative observations of the aneurysm neck		Total
		Complex	Simple	
Preoperative reconstruction estimated the condition of aneurysm neck dissection	Complex	2	1	3
	Simple	0	22	22
Total		2	23	25
The Kappa coefficient was 0.779, *P* < 0.001				

**Table 3:** Preoperative reconstruction predicts the rupture site of the aneurysm and compares it with the actual situation during surgery.

		Intraoperative aneurysm rupture site conditions		Total
		Absent	Present	
Preoperative imaging reconstruction for anticipating aneurysm rupture sites	Absent	2	4	6
	Present	3	16	19
Total		5	20	25
The Kappa coefficient was 0.186, *P* = 0.349				

**Table 4:** Preoperative reconstruction anticipates the perforating arteries near the aneurysm and compares it with the actual situation during surgery.

		Intraoperative perforating artery correspondence		Total
		Absent	Present	
Preoperative reconstruction for assessing perforating arteries near the aneurysm body	Absent	15	3	18
	Present	1	6	7
Total		16	9	25
The Kappa coefficient was 0.635, *P* < 0.001				

**Table 5:** Preoperative reconstruction predicts the stenosis of the aneurysm-bearing artery and compares it with the actual situation during surgery.

		Intraoperative actual stenosis of the parent artery carrying the aneurysm		Total
		No stenosis	Stenosis present	
Preoperative anticipation of the degree of parent artery stenosis carrying the aneurysm	No senosis	20	0	20
	Stenosis present	1	4	5
Total		21	4	25
The Kappa coefficient was 0.865, *P* < 0.001				

### Follow-up outcomes, intraoperative and postoperative complications, and prognosis

Postoperative head CT scans within 24 h revealed no new hemorrhages, and head CTA examinations within seven days postoperatively showed no recurrence of aneurysms. One case of intraoperative aneurysm re-rupture occurred with an anterior communicating artery aneurysm. Preoperative CTA reconstruction suggested that dissecting the aneurysm neck would be challenging. Despite careful exploration along the ipsilateral A1 segment, re-rupture and bleeding occurred. After temporary occlusion of the ipsilateral A1 segment, the aneurysm neck was successfully dissected and clipped. In one case, a follow-up examination indicated an infarction in the distal region of the parent artery, occurring in a patient with a ruptured anterior communicating artery aneurysm. Preoperatively, stenosis of the parent artery was anticipated, and after clipping, the aneurysm clip was adjusted repeatedly. Despite the intraoperative application of a papaverine-soaked dressing, cerebral vasospasm was observed, ultimately leading to an infarction in the frontal and parietal lobes.

### Typical case illustration

**Figure 1:**
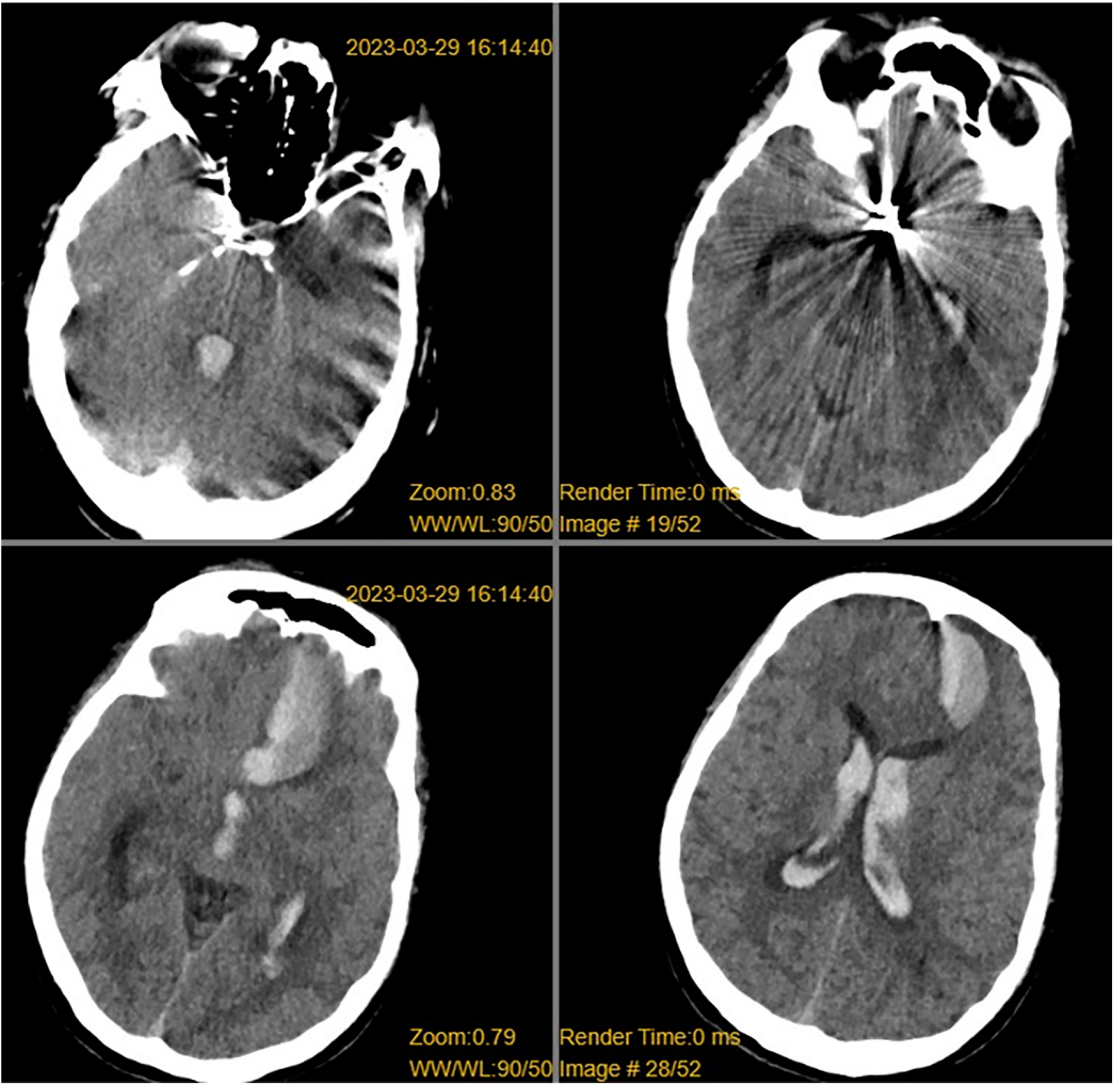
CT revealed a left frontal lobe hematoma, intraventricular hemorrhage, subarachnoid hemorrhage, and metallic artifacts in the sellar region.

**Figure 2:**
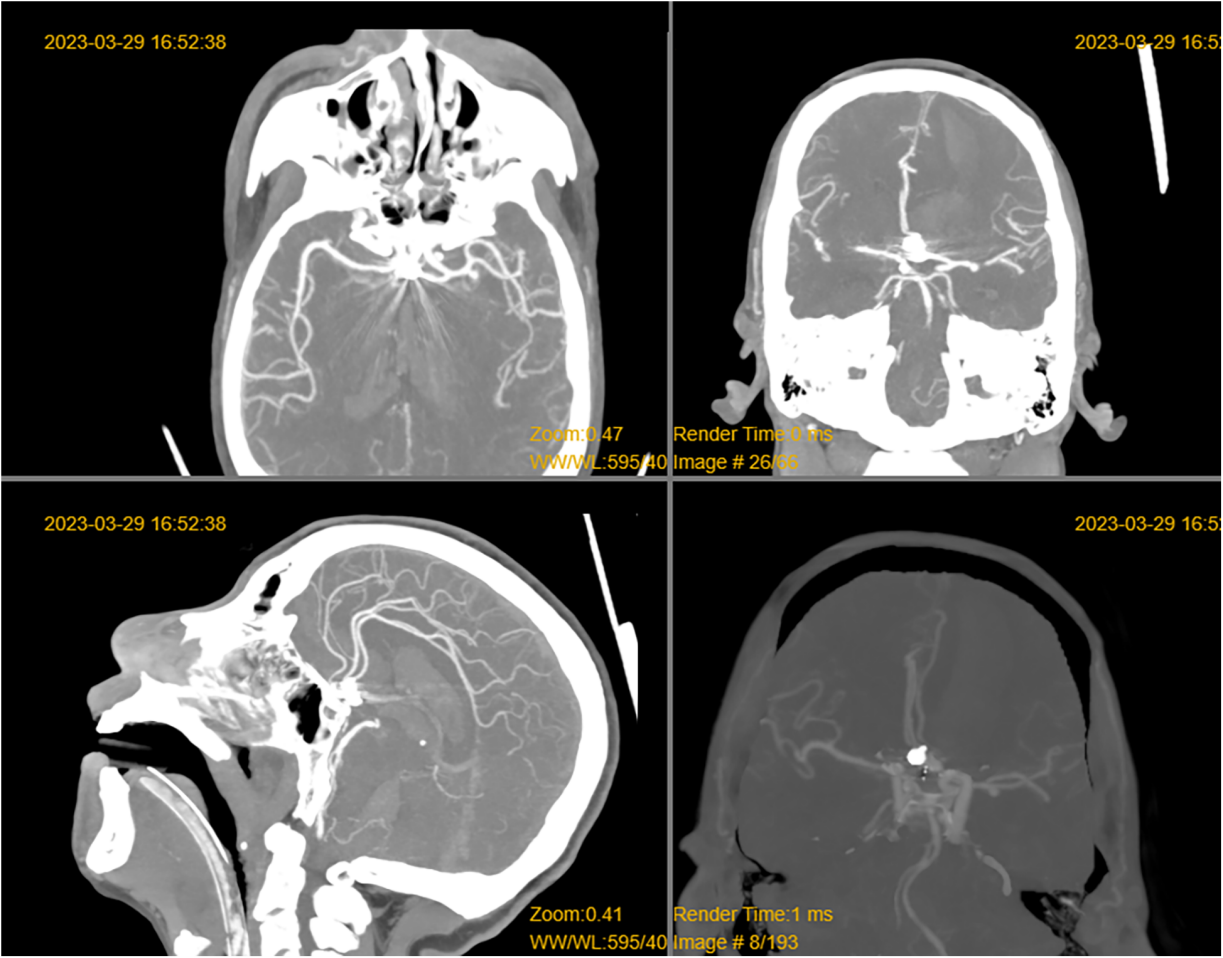
Horizontal, coronal, and sagittal reconstructions of the CTA examination.

**Figure 3:**
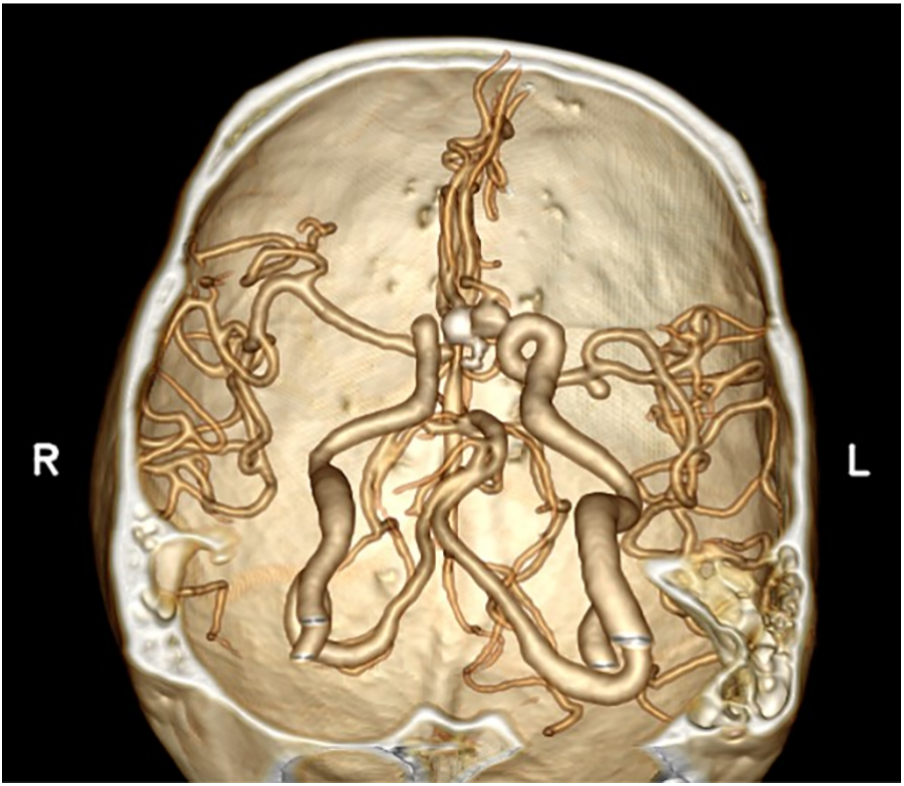
CTA examination on the imaging workstation.

**Figure 4:**
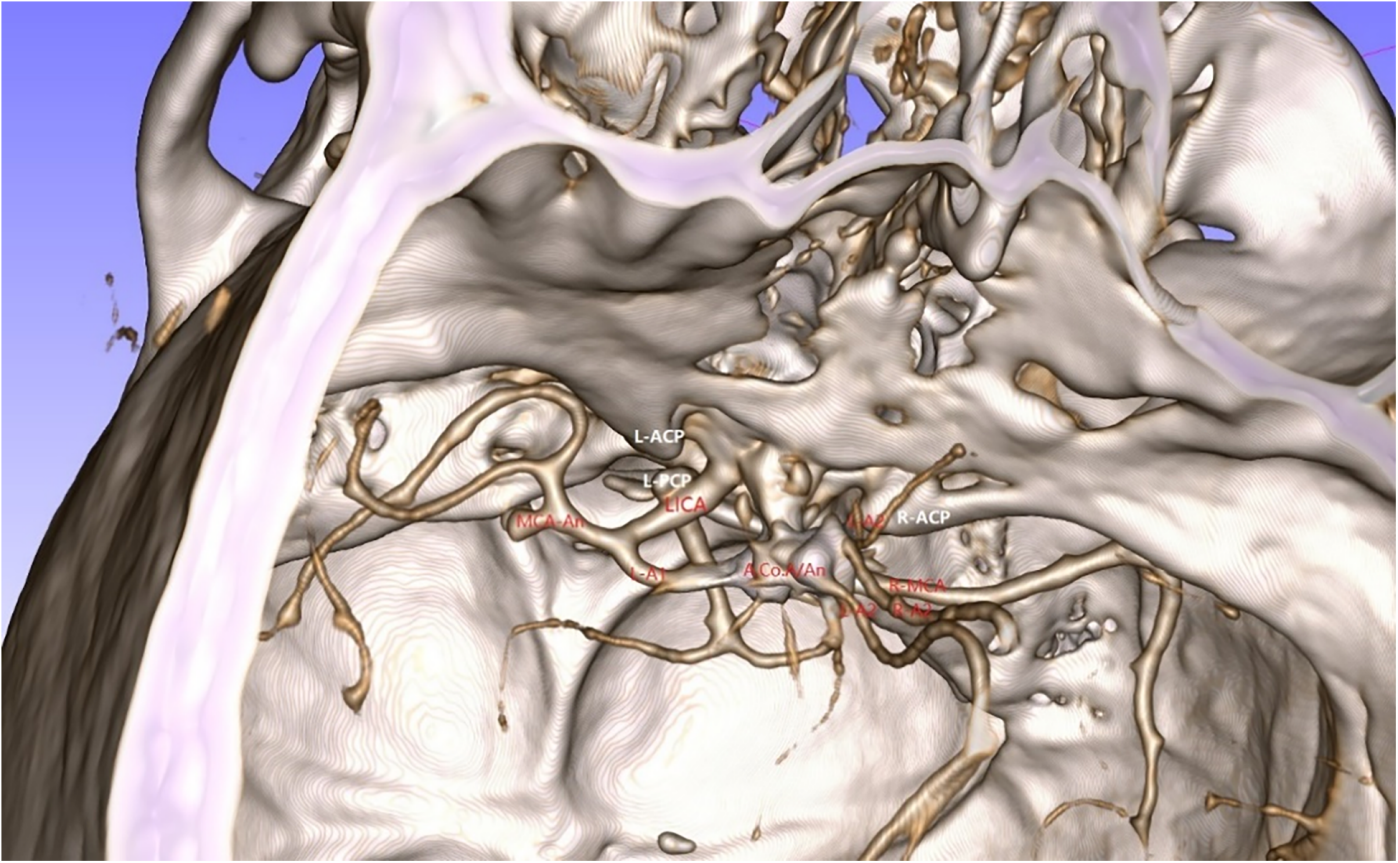
Reconstruction using combined 3D-Slicer software.

**Figure 5:**
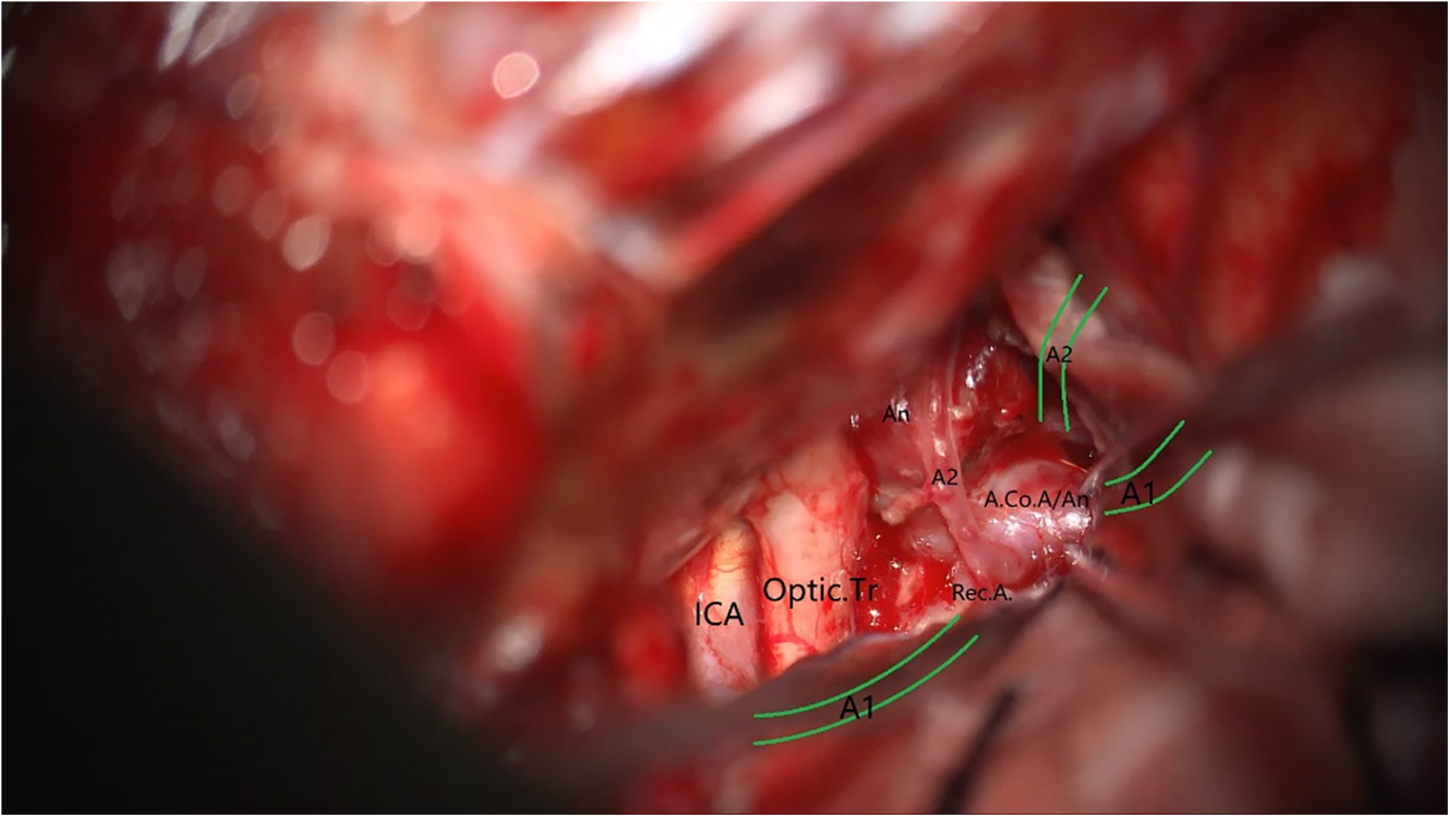
Intraoperative view under the microscope.

A 49-year-old male was admitted to the hospital due to sudden loss of consciousness for 1 h. He had a history of interventional treatment for an anterior communicating artery aneurysm without follow-up. Physical examination revealed a comatose state with a Glasgow Coma Scale (GCS) score of 5 and Hunt-Hess grade 4. A cranial CT scan indicated extensive subarachnoid hemorrhage and a left frontal hematoma (Figure 1). Cranial CTA showed metallic artifacts in the region of the anterior communicating artery (Figure 2). AI reconstruction of the CTA images on the imaging workstation highlighted possible aneurysms with concurrent vascular caliber distortions (Figure 3). Upon rotation, the anterior communicating artery interventional material created radial artifacts affecting the A1 segment of the anterior cerebral artery, leading to poor visualization. A suspicious aneurysmal protrusion was observed at the anterior communicating artery, and an aneurysm at the M1 segment of the left middle cerebral artery. Using DICOM data, reconstruction with Slicer allowed for the differentiation of metallic radial artifacts from aneurysms by adjusting the rotation angle and rendering degree. The anterior communicating artery aneurysm was noted to protrude anteriorly and superiorly, measuring approximately 8 mm in diameter; an aneurysm at the bifurcation of the M1 segment of the left middle cerebral artery, measuring about 5 mm in diameter, was also observed. Further 3D Slicer reconstruction simulated the intraoperative situation via a left pterional approach. The recurrent anterior communicating artery aneurysm suggested the possibility of an interhemispheric fissure approach and a pterional approach. Considering the hemorrhage, the anterior communicating artery aneurysm was identified as the culprit, with the aneurysm protruding anteriorly and superiorly. The left pterional approach was more favorable for clipping, and it also allowed for clipping of the middle cerebral artery aneurysm. The patient's anterior communicating artery aneurysm had a challenging neck reconstruction, with three A2 segments of the anterior cerebral artery seemingly visible distally, indicating the need for careful dissection of the aneurysm neck and attention to perforating branches and distal parent artery branches near the recurrent aneurysm (Figure 4). Following the surgical plan, a left pterional approach was utilized. Based on the angles simulated from the reconstructed images, after thoroughly drilling away the frontal bone, part of the temporal bone was also removed to facilitate clipping of the ipsilateral middle cerebral artery aneurysm. Intraoperatively, the anterior communicating artery aneurysm was clipped first. The anticipated difficulty in dissecting the aneurysm neck was associated with metallic artifacts; however, by analyzing the distal branches from the preoperative rotational view, the aneurysm neck and the A2 segment vessels of the anterior cerebral artery near the aneurysm body were accurately isolated, leading to successful clipping of the aneurysm (Figure 5). Subsequently, the ipsilateral middle cerebral artery aneurysm was clipped. Postoperative CTA examination revealed good clipping of both aneurysms, with no evidence of recurrent bleeding. At the 3-month follow-up, the Glasgow Outcome Scale (GOS) score was 5, indicating satisfactory recovery, and the patient underwent cranioplasty. The CTA examination at 3 months showed no signs of aneurysm recurrence.

## Discussion

Interventional treatment, characterized by minimal invasiveness and fewer perioperative complications, has gradually become the main treatment method for intracranial aneurysms. However, for aneurysms in the anterior circulation, or those with superficial locations due to rupture and associated intracerebral hematoma or brain herniation, open clipping remains an important therapeutic approach. To improve the suitability of aneurysms for open clipping and reduce perioperative complications such as bleeding, infection, and parent artery stenosis, more individualized and precise surgical techniques are required. Therefore, a clear preoperative diagnosis and multimodal imaging reconstruction of aneurysm details to assist in precise clipping during surgery can help to leverage strengths and mitigate weaknesses (3, 4). Image fusion and three-dimensional reconstruction not only display the aneurysm and surrounding vessels but also help to illustrate the relationships between the skull, brain parenchyma, and hematoma that are essential during surgery. Adequate preoperative time allows the surgeon to repeatedly and accurately simulate the operation, compensating for the long learning curve required for open clipping surgery.

### Advantages of CTA combined with 3D-slicer reconstruction over conventional CTA imaging

Compared to interventional treatments where the gold standard DSA examination can clearly delineate the location, size, and neck characteristics of an aneurysm, many cases that are candidates for open clipping surgery are admitted urgently due to ruptured aneurysm bleeding. We are aware that DSA is the diagnostic method of choice, especially for clipped aneurysms. In this group of patients, CTA performed shortly after aneurysm repair shows complete obliteration of the clipped aneurysm. This realization dawned on us that, in patients with ruptured intracranial aneurysms, the rapid progression of their condition precludes the systematic performance of DSA or MRA examinations, leaving CTA as the fastest available option. Before microsurgical clipping of an aneurysm, a comprehensive and clear understanding of its morphology, size, position, and parent artery is necessary to anticipate intraoperative neck dissection, perforating arteries, and potential rupture and bleeding. In clinical practice, the medical three-dimensional imaging of patients is often processed by radiologists using workstations attached to imaging machines or AI reconstruction, focusing on making a diagnosis. However, clinical specialists have different considerations; while diagnosing, they also contemplate the choice of treatment methods, and details that may seem minor could be equally important for patient care (5, 6). Slicer software was used to reconstruct imaging information, allowing radiologists to make preoperative predictions. These predictions then was compared with what the surgical team observes under the microscope, especially with the aid of fluorescence imaging, to identify parts with high consistency in predictive accuracy, thereby assisting in the precise surgical clipping of aneurysms. The radiology team includes surgeons with surgical experience, which enables the reconstruction of imaging data in the surgical position, providing more meaningful guidance. In the workstation reconstruction data of this group of patients, there were cases where the anterior cerebral artery was unclear, and the diagnosis of an anterior communicating artery aneurysm was inconclusive. The main reason was the artifacts from the skull base bone and previous interventional materials, which affected the diagnosis of the aneurysm. Additionally, this patient had a middle cerebral artery aneurysm, and reconstructions that did not include the skull base bone and were limited in deflection angles also impacted further decision-making by physicians. However, the patient's clinical manifestations and CT scans showing intracerebral hematoma indicated to the clinicians the need to focus on the anterior communicating artery and anterior cerebral artery. By reconstructing and segmenting the bone, adjusting the rendering degree, and removing the artifacts from interventional materials, the aneurysm and its parent artery were clearly displayed. Anticipating the adhesion of the aneurysm neck and perforating arteries also provided better guidance for the selection of subsequent surgical approaches.

The Disadvantages of CTA Over DSA in the Diagnosis of Intracranial Aneurysms Include: (1) The posterior communicating artery and the non-dominant A1 segment of the anterior cerebral artery may appear under-displayed due to their slender nature, creating a false impression of arterial protrusion; (2) Intracranial hematoma compression or intracranial hypertension leading to unclear visualization of the aneurysm; (3) The aneurysm's proximity to the skull base and severe obstruction by bone. To mitigate these issues, a thorough understanding of microanatomy of the brain, the pathogenesis, and the rules of aneurysm formation and growth is necessary for purposeful reconstruction, which is where the rendering, reconstruction, and segmentation capabilities of Slicer software excel. The results from this case series indicate that CTA images reconstructed with Slicer software have a higher diagnostic efficiency for intracranial aneurysms, particularly in detecting aneurysms with a diameter of less than 5 mm compared to traditional imaging workstations. Preoperative reconstruction and analysis of aneurysm morphological characteristics facilitate the anticipation of intraoperative maneuvers. In practice, there was a good performance in the prediction of parent artery stenosis, aneurysm neck morphology reconstruction, and the anticipation of perforating arteries near the aneurysm. Specifically, the reconstruction effectively distinguished between hematoma, bone, and aneurysm neck, allowing for rotation to positions near the surgical approach to locate the neck and clipping pathway, which aids in the targeted separation of the aneurysm neck. The multi-angle observation of the aneurysm neck during the simulated surgical process helps surgeons avoid mistakenly clipping adjacent arterial branches (7). This reduces disturbance to vessels that may have aneurysm formation or arteriosclerosis, decreases the incidence of parent artery stenosis, and also prevents intraoperative aneurysm re-rupture (only one case in this series). Thanks to preoperative feature analysis, the clipping method was pre-planned to reduce the stenosis of the parent artery and the misclipping of perforating arteries, with three patients undergoing Drake's tandem clipping, supplemented by straight clips on the proximal end, without increasing the selection clamping time. The poor consistency in the prediction of the aneurysm rupture site is considered to be due to the large individual differences in CTA data acquisition, with the timing of the scan being particularly important for the full display of vascular details, and subsequent reconstruction unable to reveal more daughter sac details. The preoperative three-dimensional imaging observation of the aneurysm orientation is objective, but the judgment of the relationship between the daughter sac protrusions on the aneurysm and the bleeding is more subjective, with poor consistency. This indicates that future preoperative observation of aneurysm morphology should be more meticulous, and if necessary, combined with a comprehensive analysis of medical history, hematoma location, and the amount of subarachnoid hemorrhage.

### Personal experience with precise surgical planning in the guidance of surgery for this case series

Aneurysms of the anterior circulation are typically treated with pterional approaches, interhemispheric fissure approaches, or skull base approaches for open clipping surgery. Patients with deep-seated aneurysms, low preoperative scores, and associated brain tissue swelling may undergo an extended pterional approach or even an orbitozygomatic approach (8). In this case series, individualized and precise surgical planning was able to reduce unnecessary damage and anticipate and avoid intraoperative risks associated with manipulation of the aneurysm before the neck is fully exposed. The most catastrophic complication in clipping surgery is re-rupture of the aneurysm, which, if it occurs before the aneurysm neck is completely dissected or the proximal parent artery is well-exposed, can lead to massive intraoperative bleeding and poor prognosis due to the inability to rapidly achieve temporary occlusion (9). In this series, three cases were anticipated to have difficult neck dissection, and seven cases had perforating arteries close to the aneurysm neck. Simulated surgical field observation of the aneurysm neck helped surgeons anticipate and avoid disturbing the weak areas of the aneurysm body and the perforating arteries adjacent to the neck. The results indicated accurate prediction of difficult neck dissection, with a positive predictive value of 85.71% for perforating arteries, demonstrating a high level of consistency. Postoperative comparison with reconstructed images revealed a false-negative rate of 16.67%, significantly affected by poor opacification, indicating the need for repeated rendering and judgment. Additionally, excessive retraction to increase exposure can lead to injury of perforating arteries and contusion of brain tissue, causing additional neurological damage to the patient. If a routine pterional approach is used with the head positioned in a 30–60° retroflexion and contralateral rotation, it cannot be quantified or personalized, resulting in adjustments to the bed angle during surgery or excessive retraction with a brain spatula. In this case series, our preoperative reconstruction revealed significant displacement of the internal carotid artery and the proximal middle cerebral artery on the affected side compared to the healthy side, due to intracranial hematoma and brain tissue swelling. Based on a balance of the proximal parent artery, the direction of aneurysm neck clipping, and the clipping pathway after sylvian fissure dissection, a 15-degree retroflexion and 15-degree contralateral rotation were determined to be the optimal positions for clipping of middle cerebral artery aneurysms and anterior communicating artery aneurysms. For posterior communicating artery aneurysms, further assessment of the neck position is required, with a 30-degree contralateral rotation being more beneficial for exposure of the origin of the posterior communicating artery and exploration of important perforating arteries in the third interval. While clipping the aneurysm, efforts should be made to maintain patency of the parent artery. In this series, the negative predictive accuracy for parent artery stenosis was 100%, the positive predictive value was 80%, and the false-positive rate was 20%, indicating a relatively accurate prediction of parent artery stenosis. During surgery, anticipating parent artery stenosis helps in the early selection of appropriate clips and planning of clipping methods. Understanding the three-dimensional morphology of the parent artery, rendering to determine arterial plaques, and simulating clipping are crucial. In this series, one case with a false positive result for the parent artery stenosis required a clipping method different from the preoperative simulation (10, 11). The compression by a hematoma adjacent to the aneurysm neck suggested a small-caliber parent artery and indicated stenosis. However, upon hematoma removal during surgery, the parent artery was found to be without significant stenosis. Empirical drilling of the sphenoid ridge may lead to excessive removal. Although skull base bone can be sacrificed compared to brain tissue, more drilling and increased exposure are also good methods to reduce retraction (12–14). In this case series, we observed that for middle cerebral artery aneurysms, exposure of the inferolateral aspect of the frontal operculum can unfold the direction of aneurysm neck clipping; anterior communicating artery aneurysms require drilling of the sphenoid ridge to the orbital lateral wall cortex, and drilling along the inner surface of the skull to the base of the frontal region, reducing retraction and facilitating hematoma clearance at the base of the frontal lobe and lamina terminalis fenestration; some patients with posterior communicating artery aneurysms may require drilling of the sphenoid ridge to the superior orbital fissure, suspending the dura mater to increase exposure of the second and third intervals, facilitating the placement of aneurysm clips. If patients have ruptured bleeding combined with intracerebral hematoma, considering the removal of the bone flap for decompression after hematoma evacuation requires the removal of more skull base bone. However, for patients with a short duration of onset, moderate hematoma volume, and slightly older age, performing clipping surgery within the above exposure range and returning the bone flap postoperatively can significantly reduce skull base bone defects.

### Technical limitations and future prospects

Currently, there is a growing number of reports on the use of 3D Slicer for post-processing of imaging in intracranial tumors and cerebrovascular diseases. Targeted reconstruction of lesions and surrounding structures can assist in precise intraoperative localization and serves as an important supplement to preoperative planning (15, 16). The conditions required for using Slicer for reconstruction are minimal, making it easy to popularize and implement. A computer equipped with an independent graphics card, two doctors, and a small amount of time—typically 10–15 min before surgery—to perform the image reconstruction, saving the reconstructed images or animations for use during the operation. However, this case series has a limited sample size and lacks support from large datasets, only yielding some simple patterns from preliminary application methods. Therefore, whether the combination of CTA and 3D-Slicer imaging reconstruction can significantly improve outcomes, especially for patients with higher-grade intracranial aneurysms, remains to be explored. With the widespread adoption of electronic imaging systems such as PACS in hospitals, it is not difficult for neurosurgeons to obtain DICOM data for further processing (17). This method can be well integrated into the preoperative planning of intracranial aneurysms, and when necessary, 3D printing can be used for medical school education and clinical communication with families (18–20). It is anticipated that in the future, multimodality imaging fusion technology will aid in surgical treatments and become a focal point within the field of neurosurgery. Additionally, we have observed that while the reconstruction of CTA data is beneficial for pre-operative planning, it introduces increased metal artefacts adjacent to the aneurysm post-surgery. Even with the assistance of Slicer software, the assessment is limited to indirect judgments based on the presence or absence of bleeding or distal arterial ischemia. Therefore, post-operative evaluation may require a combination of CTA, DSA, and silent MRA to collaboratively determine the outcome.

## Conclusion

In summary, the combination of 3D-Slicer and CTA technology can accurately diagnose and assess intracranial aneurysms, anticipate important anatomical information required for open clipping surgery, plan surgical procedures, formulate comprehensive surgical plans, determine surgical approaches, analyze aneurysm characteristics for preoperative judgment, establish clipping plans, and preselect aneurysm clips, which helps to reduce surgical risks and intraoperative complications. These findings lay the foundation for further research and provide better recommendations for diagnostic and therapeutic decision-making and surgical planning.

## Data Availability

The original contributions presented in the study are included in the article/Supplementary Material, further inquiries can be directed to the corresponding author.
